# Multi-hit genetic lesions and a stress-imprinted immune transcriptome define the inflammatory pathology in ALS patients

**DOI:** 10.21203/rs.3.rs-10046801/v1

**Published:** 2026-07-10

**Authors:** Ao Mei, Dipanarine Jewett, Anahid Jewett, Kawaljit Kaur, Subramaniam Malarkannan

**Affiliations:** Medical College of Wisconsin; University of California, Los Angeles; University of California, Los Angeles; University of California, Los Angeles; Medical College of Wisconsin

**Keywords:** Amyotrophic lateral sclerosis (ALS), natural killer (NK) cells, γδ T cells, TNF-α, NF-κB, IFN-γ response, whole-genome sequencing, single-cell RNA sequencing

## Abstract

Amyotrophic lateral sclerosis (ALS) is a genetically heterogeneous neurodegenerative disease whose peripheral immune architecture remains incompletely defined. Here, we integrated whole-genome sequencing and single-cell RNA sequencing to define genomic and immune correlates of ALS. Genome-wide analysis of a monozygotic twin pair discordant for ALS pathology identified shared ALS-associated variants, as well as patient-enriched variants in genes linked to RNA metabolism, neurodegeneration, and immune inflammation, supporting a multilayered genetic architecture. Single-cell profiling of 40,484 peripheral blood mononuclear cells from three ALS patients and two healthy individuals, including 33,667 cells retained after quality control, resolved 13 immune clusters and revealed broad remodeling of the peripheral immune compartment, with relative enrichment of natural killer, mucosal-associated invariant T, and γδ T-cell populations. Across immune subsets, ALS samples exhibited inflammatory and stress-adapted transcriptomic programs, including TNF-α/NF-κB, IFN-γ, hypoxia, and ribosomal stress pathways. These data support a model in which multi-hit genetic susceptibility converges on a stress-induced immune transcriptome, marked uniquely by innate lymphocyte activation in ALS patients.

## Introduction

Amyotrophic lateral sclerosis (ALS) is a progressive and fatal neurodegenerative disease characterized by destruction of upper and lower motor neurons, leading to worsening weakness, paralysis, and respiratory failure. Approximately 10% of ALS cases are familial, whereas the majority are classified as sporadic, and even familial disease often cannot be explained by a single fully penetrant variant (1–8). Instead, increasing evidence suggests that ALS frequently reflects multilayered genetic susceptibility shaped by combinations of rare and common variants, incomplete penetrance, and modifying biological context (8). In sporadic ALS, polygenic susceptibility, rare variants, and environmental influences are all thought to contribute to disease risk and heterogeneity. Although genome-wide association and sequencing studies have identified multiple ALS-associated loci, each explains only a fraction of the overall disease burden, and the genetic basis of many cases remains unresolved (1, 3). Despite major advances in sequencing-based gene discovery, the genetic basis of disease heterogeneity and its relationship to downstream pathogenic programs remain incompletely resolved (8, 9). Therefore, defining disease-associated variants and genetic modifiers remains both biologically and clinically important (3–5).

At the mechanistic level, ALS is characterized by progressive loss of motor neuron cell bodies in the motor cortex, brainstem, and spinal cord, together with degeneration of corticospinal tracts and denervation of skeletal muscle (1, 9, 10). A key molecular hallmark of ALS is abnormal TAR DNA-binding protein 43 (TDP-43) biology, including mutation, aggregation, mislocalization, or loss of normal nuclear function (10–14). Additional major genetic contributors include mutations in *SOD1* and *FUS*, as well as hexanucleotide repeat expansions in *C9orf72* (15–19). These lesions account for a substantial proportion of familial ALS. Still, numerous additional genes have also been implicated, many of which converge on pathways involving RNA metabolism, proteostasis, mitochondrial homeostasis, DNA repair, vesicular trafficking, and axonal integrity (2, 8, 9). Together, these observations indicate that ALS is genetically heterogeneous but mechanistically convergent, with diverse upstream causes feeding into overlapping degenerative pathologies.

Beyond neuron-intrinsic pathology, growing evidence points to immune system dysregulation playing a key role in ALS. Neuroinflammation is a common feature of disease and has been classically linked to microglial activation within the central nervous system (20, 21). However, peripheral immune cells are increasingly recognized as active participants in disease biology rather than passive responders to tissue injury. CD4^+^ T cells can recognize C9orf72-derived epitopes and produce cytokines such as IL-5 and IL-10 (22–25), whereas clonally expanded antigen-experienced CD8^+^ T cells have been shown to promote neuronal injury in a murine model of ALS4 driven by *SETX* mutation (26). B-cell-based immunomodulatory approaches have shown protective effects in preclinical models and in limited clinical settings (27). NK cells have also been implicated in ALS pathogenesis, including evidence that they can mediate toxicity toward motor neurons carrying the *hSOD1G93A* mutation (28). In addition, monocytes and neutrophils may amplify inflammatory injury and contribute to disease progression (29). Collectively, these findings support the concept that ALS involves a broader neuroimmune axis in which peripheral and tissue-localized immune responses may shape disease trajectory and severity.

Recent transcriptomic studies have begun to refine this view by identifying disease-associated molecular programs in both neural and immune compartments. Analyses of human motor and prefrontal cortex in ALS and frontotemporal lobar degeneration have identified increased NEFL expression associated with axonal degeneration and potential prognostic value (30). Single-cell studies of ALS-relevant neural models have further highlighted mitochondrial dysfunction and stress-response abnormalities as central features of motor neuron vulnerability. In parallel, single-cell RNA sequencing of peripheral immune cells has suggested substantial immune remodeling in ALS, including expansion of a CD56^Dim^ NK-cell subset in PBMCs and the presence of unusual stem-like CD3^+^CD16^+^CD8^+^ T-cell states that distinguish patients from healthy controls (31, 32). Together, these studies indicate that ALS-associated molecular dysregulation extends beyond motor neurons and can be detected in circulating immune cells. Still, the immune populations driving disease-associated inflammatory programs are yet to be fully defined.

We previously found that ALS patients had higher percentages of NK cells, along with increased levels of granzyme B and perforin in these cells (33). Activated CD8^+^ T cells from ALS patients showed higher IFN-γ spots or secretion compared to healthy controls, as well as elevated expression of CD28 and CCR7, but lower expression of IFN-γ receptors (34). In addition to increased IFN-γ and TNF-α, CD8^+^ T cells in patients had much higher levels of granzyme B at the single-cell level. ALS patients also showed significantly higher serum levels of G-CSF, eotaxin, IP10, Rantes, VEGF, IL-12p70, IL-6, IL-17a, IL-10, IFN-γ, and TNF-α, which correlated with increased cytokine secretion from PBMCs, NK cells, and CD8^+^ T cells, highlighting the key role of IFN-γ and TNF-α in ALS pathogenesis (33, 35). Both pro- and anti-inflammatory cytokines were elevated, suggesting overall heightened immune activation. T_Reg_ analysis revealed higher percentages of Foxp3^+^ populations in PBMCs. While ALS patients had fewer expanded T_Reg_ after naïve CD4^+^ T cell differentiation, these T_Reg_ secreted more IL-10 than those from healthy controls, with IL-10 secretion exceeding that of IFN-γ in T_Reg_ (33, 34).

In the present study, we used whole-exome sequencing (WES) and single-cell RNA sequencing to define genetic variation and peripheral immune remodeling in ALS. We first analyzed monozygotic twin brothers discordant for ALS to identify shared and patient-enriched variants in genes previously linked to ALS pathogenesis. Next, we analyzed PBMCs from three ALS patients and two healthy controls at single-cell resolution to characterize disease-associated transcriptional states across lymphoid and myeloid compartments. Our results revealed broad immune remodeling, including relative enrichment of innate-like lymphocyte populations and activation of inflammatory transcriptional programs involving TNF-α/NF-κB, IFN-γ, and hypoxia-associated pathways across multiple immune subsets. Notably, NK cells and γδ T cells exhibited the strongest chemokine- and cytokine-associated inflammatory signatures, supporting a model in which multilayered genetic susceptibility converges on a stress-imprinted immune transcriptome in ALS. Overall, these findings identify a systemic inflammatory immune state in ALS and demonstrate NK cells and γδ T cells as prominent disease-associated peripheral immune populations.

## Results

### Genomic analysis of discordant monozygotic twins identifies shared and patient-enriched ALS-associated variants

To define genetic features associated with ALS manifestation in a shared hereditary background, we performed whole-exome sequencing on PBMC-derived genomic DNA from monozygotic twin brothers discordant for ALS. Because monozygotic twins share the same genetic background, this design provides an opportunity to distinguish shared ALS-associated variants, which may reflect inherited susceptibility, from twin-specific variants, which may contribute to disease penetrance, modifier effects, or resilience. We analyzed the WGS data and found that the overall variant landscape was not compatible with a single deterministic mutation. Instead, it supported a shared genetic background risk together with additional twin-specific gene alterations (**Table 1**) (1–7).

A first category comprised ALS-twin-specific variants in genes with established or plausible relevance to ALS biology. These included multiple *TARDBP* missense variants as well as variants in *ERBB4*, *PRF1*, *ANG*, *SPG11*, *ATXN2L*, and *FUS* genes (**Table 1**). The presence of several *TARDBP* variants is notable given the central role of TDP-43 dysfunction in ALS pathogenesis, including altered localization, aggregation, and nuclear dysfunction (10–14). Likewise, variants in *FUS* and *ANG* are consistent with prior studies linking RNA dysregulation, stress responses, and neurodegeneration to ALS susceptibility (8, 12, 15–19). In the context of our immune analyses, the affected twin also carried a *PRF1* gene missense variant, suggesting that cytotoxic immune pathways may intersect with the disease-associated genetic architecture. However, functional validation of this missense mutation will be required to establish its significance. A second category comprised healthy-twin-specific variants, including splice-site variants in *C9orf72* and *HNRNPA1* genes (**Table 1**). This observation underscores an important principle: the presence of a variant in a canonical ALS-associated gene does not, by itself, predict clinical disease. Instead, our observation argues against a single-hit model. This is especially relevant in ALS, where both familial and sporadic forms are increasingly understood to arise from heterogeneous combinations of high-impact mutations, lower-penetrance alleles, and disease-modifying factors (1–7, 22–25).

A third category comprised shared variants present in both twins, including those in *ALS2*, *NEK1*, *PRPH2*, Fig. 4, *SIGMAR1*, *SETX*, *OPTN*, *ATXN2*, *TRPM7*, *SARM1*, *UNC13A*, and *NEFH* genes (**Table 1**). Several of these genes are well linked to neuronal maintenance, axonal integrity, RNA metabolism, vesicular trafficking, stress signaling, or neurodegenerative vulnerability (2). Importantly, multiple genes with shared variants also contained additional twin-specific variants, further strengthening a combinatorial model. For example, ALS2 contained shared splice-site and missense variants together with an additional ALS-twin-specific missense variant, whereas *PRPH2*, Fig. 4, *SETX*, *TRPM7*, *UNC13A*, and *NEFH* genes also showed shared background variants accompanied by distinct ALS-twin-specific or healthy-twin-specific alterations (**Table 1**). Collectively, the exome data support a multi-hit genetic model in which shared variants likely represent a permissive background, whereas patient-enriched variants may shift the balance toward disease pathology.

### Single-cell transcriptomic profiling identifies preserved and selective immune remodeling in ALS.

**Figure 1:**
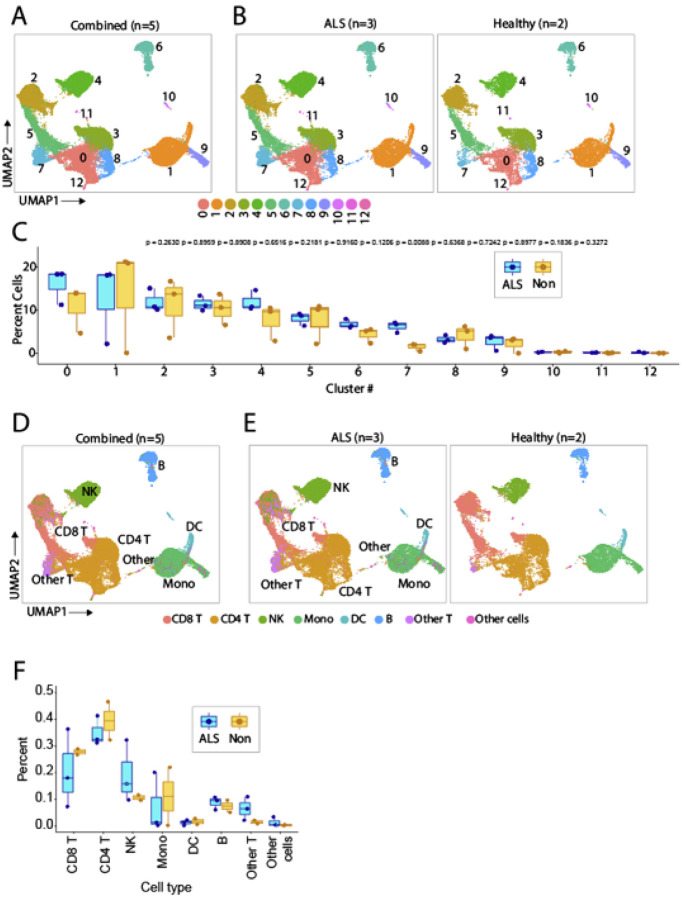
Single-cell transcriptomic profiling identifies major PBMC populations in ALS and healthy controls. (**A**) Uniform manifold approximation and projection (UMAP) of all quality-controlled peripheral blood mononuclear cells (PBMCs) from the combined dataset (n = 5; ALS, n = 3; healthy, n = 2), showing 13 transcriptionally distinct clusters identified by unsupervised analysis. Cluster numbers are indicated. (**B**) UMAPs of PBMCs from ALS and healthy samples are shown separately, demonstrating the representation of all major clusters in both groups. (**C**) Box plots showing the percentage of cells within each cluster in ALS and healthy samples. P values are shown above the corresponding comparisons. (**D**) Broad lineage annotation of the combined PBMC dataset based on canonical marker genes and SingleR classification, identifying CD8 T cells, CD4 T cells, natural killer (NK) cells, monocytes, dendritic cells (DCs), B cells, and other minor populations. (**E**) Broad lineage annotation shown separately for ALS and healthy samples. (**F**) Box plots showing the percentage of broad immune lineages in ALS and healthy samples.

To determine whether this genetically complex background was accompanied by coordinated remodeling of the peripheral immune compartment, we performed single-cell RNA sequencing on PBMCs from three ALS patients and two unrelated healthy controls, including the affected monozygotic twin analyzed by exome sequencing. The 10x Genomics Chromium platform yielded 40,484 captured cells across five samples. After quality control filtering, 33,667 high-quality cells were retained, including 20,519 from patients with ALS and 13,148 from healthy controls. Unsupervised clustering identified 13 transcriptionally distinct clusters across the combined dataset (Fig. 1A).

When ALS and healthy samples were visualized separately, all major clusters remained represented in both groups (Fig. 1B), indicating that ALS did not generate qualitatively new transcriptomically distinct subsets, but instead altered the distribution and transcriptional state of existing immune populations. At the cluster level, several populations showed quantitative differences between ALS and healthy samples. However, only cluster 7 reached statistical significance in the current cohort (Fig. 1C). Annotation using canonical markers and SingleR identified the expected major PBMC lineages, including CD4^+^ T cells, CD8^+^ T cells, NK cells, B cells, dendritic cells, monocytes, and minor T-lineage populations (Fig. 1D–F) (36, 37). Broad lineage proportions remained largely comparable between ALS and healthy controls, suggesting that the dominant immune signal in ALS reflects selective remodeling of subset composition and cell state rather than wholesale replacement of major PBMC lineages.

### ALS is associated with relative enrichment of innate-like lymphocyte populations.

**Figure 2:**
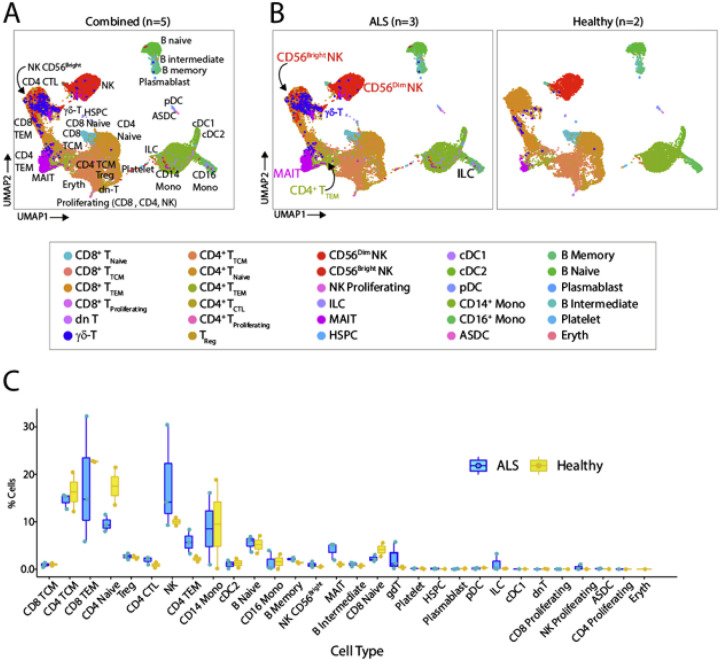
High-resolution annotation reveals selective enrichment of innate-like lymphocyte populations in ALS. (**A**) UMAP of the combined PBMC dataset annotated at higher resolution, identifying CD4 and CD8 T-cell subsets, regulatory T cells (Treg), double-negative T cells (dnT), γδ T cells, mucosal-associated invariant T (MAIT) cells, CD56^Dim^ NK cells, CD56^Bright^ NK cells, proliferating NK cells, B-cell subsets, plasmablasts, monocyte subsets, dendritic-cell subsets, and additional minor populations. (**B**) High-resolution UMAPs shown separately for ALS and healthy samples, highlighting redistribution of selected innate-like lymphocyte populations, including CD56^Dim^ NK, CD56^Bright^ NK, MAIT, and innate lymphoid cell (ILC)-like populations. (**C**) Box plots showing the percentage of annotated immune subsets in ALS and healthy samples.

We next annotated lymphoid and myeloid subsets at higher granularity. This analysis identified CD4^+^ or CD8^+^ central memory and effector memory T cells, CD4^+^ regulatory T cells, double-negative T cells, γδ T cells, MAIT cells, naïve and memory B cells, plasmablasts, CD56^Dim^ NK cells, CD56^Bright^ NK cells, proliferating lymphocytes, CD14^+^ and CD16^+^ monocytes, pDCs, cDC1, cDC2, and additional minor populations (Fig. 2A) (36, 37). Comparison of subset frequencies revealed selective remodeling of the ALS immune compartment, with a relative enrichment of innate-like lymphocyte populations rather than broad expansion of conventional adaptive lineages. Among the most prominent changes were increased representation of CD56^Dim^ NK cells, CD56^Bright^ NK cells, MAIT cells, and ILC-like populations in ALS samples (Fig. 2B, C). In parallel, the CD4 compartment redistributed from naïve-like to effector/memory-like states, with reduced naïve CD4^+^ T cells and increased CD4^+^ effector memory T cells. By contrast, major B-cell and conventional CD8 T-cell compartments did not show comparably large compositional shifts. These findings are consistent with recent work implicating NK-cell remodeling in sporadic ALS and extend that framework by suggesting that ALS preferentially enriches cell types poised for rapid cytokine, chemokine, and cytotoxic responses (37, 38).

### CD4^+^ and CD8^+^ T cells acquire inflammatory and stress-adapted transcriptional programs in ALS.

**Figure 3:**
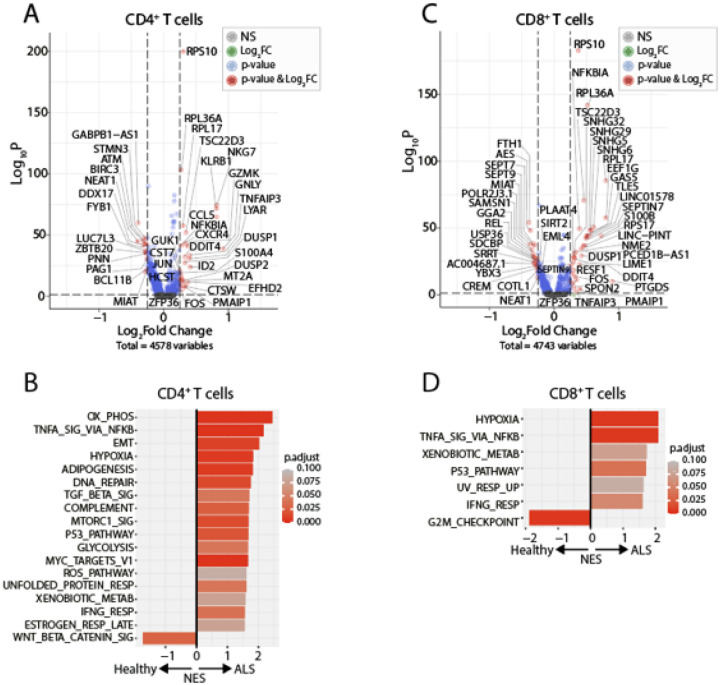
CD4^+^ and CD8^+^ T cells acquire inflammatory and stress-associated transcriptional programs in ALS. (**A**) Volcano plot showing differentially expressed genes in CD4^+^ T cells from ALS versus healthy samples. Representative upregulated and downregulated genes are labeled. (**B**) Gene set enrichment analysis (GSEA) of CD4^+^ T-cell transcriptional programs. Positive normalized enrichment score (NES) values indicate pathways enriched in ALS, whereas negative NES values indicate pathways enriched in healthy controls. (**C**) Volcano plot showing differentially expressed genes in CD8^+^ T cells from ALS versus healthy samples. Representative upregulated and downregulated genes are labeled. (**D**) GSEA of CD8^+^ T-cell transcriptional programs. Positive NES values indicate pathways enriched in ALS, whereas negative NES values indicate pathways enriched in healthy controls.

Although broad T cell abundance was not markedly altered, both CD4^+^ and CD8^+^ T cells exhibited clear disease-associated transcriptional remodeling. In CD4^+^ T cells, ALS-associated genes included RPS10, *RPL36A*, *RPL17*, *TSC22D3*, *NKG7*, *KLRB1*, *GZMK*, *GNLY*, *TNFAIP3*, *CCL5*, *NFKBIA*, *CXCR4*, *ID2*, *CTSW*, and *PMAIP1* transcripts (Fig. 3A). This profile is consistent with a coordinated shift toward inflammatory activation, heightened translational demand, and partial acquisition of innate-like effector features. Increased expression of *CCL5* and *CXCR4* transcripts suggests an activated and migratory phenotype. In contrast, upregulation of *GZMK*, *NKG7*, *GNLY*, *CTSW*, and *KLRB1*/*CD161* transcripts suggests remodeling toward a cytokine-competent effector state rather than a quiescent helper phenotype (39–42). Gene-set enrichment analysis reinforced this interpretation. CD4^+^ T cells from ALS samples were enriched for TNF-α signaling via NF-κB, hypoxia, EMT, glycolysis, mTORC1 signaling, reactive oxygen species, and unfolded protein response pathways (Fig. 3B). These findings indicate a broader state of chronic inflammatory and metabolic adaptation. This is notable in ALS, where proteostasis failure, RNA dysregulation, and stress signaling are central pathogenic features in neurons, suggesting that peripheral T cells may also experience a disease-conditioned stress environment (10, 11, 43).

CD8^+^ T cells displayed a related but distinct transcriptional program. ALS-associated genes included *RPS10*, *NFKBIA*, *RPL36A*, *TSC22D3*, *SNHG32*, *SNHG29*, *SNHG5*, *SNHG6*, *RPL17*, *EEF1G*, *GAS5*, *LINC01578*, *SEPTIN7*, *S100B*, *FOS*, *PTGDS*, and *PMAIP1* transcripts (Fig. 3C). In addition to inflammatory and stress-response genes, this profile highlights altered noncoding RNA expression, particularly the *SNHG* family and *LINC01578*. Because ALS pathobiology is deeply linked to perturbed RNA metabolism and RNA-binding protein function, these findings raise the possibility that post-transcriptional dysregulation extends into circulating immune cells rather than remaining restricted to neurons (44, 45). Pathway analysis in CD8^+^ T cells showed enrichment of hypoxia, TNF-α/NF-κB signaling, xenobiotic metabolism, p53 pathway, UV response, and IFN-γ response, whereas G2M checkpoint was relatively enriched in controls (Fig. 3D). Together, the T cell data support a model in which ALS reprograms both conventional helper and cytotoxic T cell compartments toward inflammatory, stress-adapted states.

### B cells, monocytes, and dendritic cells display lineage-specific inflammatory remodeling in ALS.

**Figure 4:**
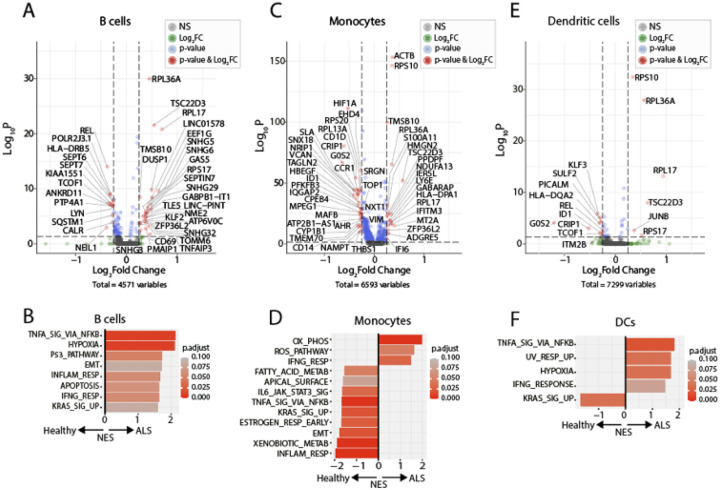
B cells, monocytes, and dendritic cells show lineage-specific transcriptional remodeling in ALS. (**A**) Volcano plot showing differentially expressed genes in B cells from ALS versus healthy samples. Representative genes are labeled. (**B**) GSEA of B-cell transcriptional programs. Positive NES values indicate pathways enriched in ALS. (**C**) Volcano plot showing differentially expressed genes in monocytes from ALS versus healthy samples. Representative genes are labeled. (**D**) GSEA of monocyte transcriptional programs. Positive NES values indicate pathways enriched in ALS, whereas negative NES values indicate pathways enriched in healthy controls. (**E**) Volcano plot showing differentially expressed genes in dendritic cells from ALS versus healthy samples. Representative genes are labeled. (**F**) GSEA of dendritic-cell transcriptional programs. Positive NES values indicate pathways enriched in ALS.

In B cells, ALS-associated transcripts included *RPL36A*, *TSC22D3*, *RPL17*, *LINC01578*, *EEF1G*, *SNHG5*, *SNHG6*, *GAS5*, *RPS17*, *SEPTIN7*, *CD69*, *PMAIP1*, and *TNFAIP3* transcripts (Fig. 4A). This profile suggests that B cells in ALS are not quiescent but instead occupy a state of compensatory immune regulation. Upregulation of *CD69* is consistent with recent activation, whereas *TNFAIP3* and *TSC22D3* suggest induction of pathways that may restrain excessive inflammatory signaling. Gene-set enrichment analysis supported this interpretation, showing enrichment for TNF-α/NF-κB signaling, hypoxia, the p53 pathway, EMT, the inflammatory response, apoptosis, and the IFN-γ response in ALS B cells (Fig. 4B).

The myeloid compartment showed lineage-specific remodeling rather than a uniform inflammatory pattern. In monocytes, ALS-associated genes included *ACTB*, *RPS10*, *TMSB10*, *RPL36A*, *S100A11*, *HMGN2*, *TSC22D3*, *RPL17*, *IFITM3*, *MT2A*, and *ADGRE5* transcripts, indicating increased cytoskeletal activity, translational capacity, and stress adaptation (Fig. 4C). Several homeostatic or differentiation-linked genes, including *HIF1A*, *G0S2*, *CCR1*, *VCAN*, *HBEGF*, and *NRIP1* transcripts, were reduced. Pathway analysis suggested that ALS monocytes are not simply shifted toward a strongly TNF-dominant inflammatory state; rather, they showed relative enrichment of oxidative phosphorylation, reactive oxygen species, and IFN-γ response, whereas pathways such as fatty-acid metabolism, IL6-JAK-STAT3 signaling, TNF-α/NF-κB signaling, EMT, and inflammatory response were relatively higher in controls (Fig. 4D).

Dendritic cells aligned more closely with the inflammatory lymphoid program. ALS-associated dendritic-cell genes included *RPS10*, *RPL36A*, *RPL17*, *TSC22D3*, *JUNB*, and *RPS17* transcripts. In contrast, genes such as *G0S2*, *CRIP1*, *TCOF1*, and *ITM2B* transcripts were decreased (Fig. 4E). Their enriched pathways included TNF-α/NF-κB signaling, UV response, hypoxia, and IFN-γ response (Fig. 4F). Together, these findings suggest that myeloid remodeling in ALS is not monolithic: dendritic cells appear more directly aligned with inflammatory activation, whereas monocytes occupy a more mixed metabolic and stress-adapted state.

### NK, γδ T, and MAIT cells define an innate-like inflammatory axis in ALS.

**Figure 5:**
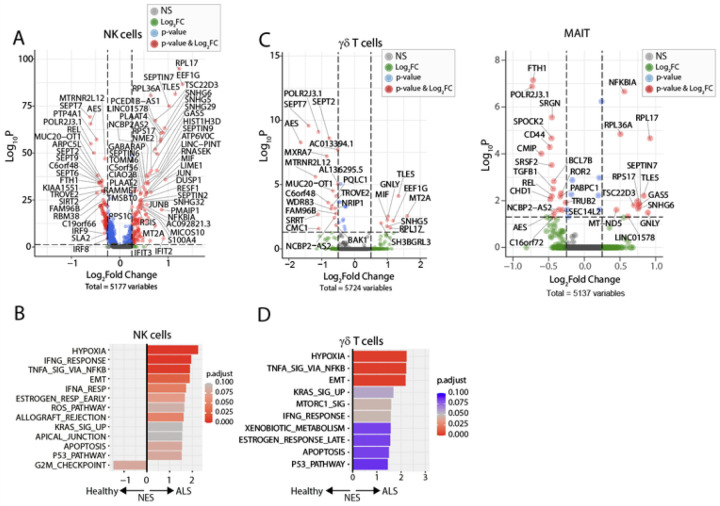
Innate-like lymphocyte populations define a prominent inflammatory axis in ALS. (**A**) Volcano plot showing differentially expressed genes in NK cells from ALS versus healthy samples. Representative genes are labeled. (**B**) GSEA of NK-cell transcriptional programs, showing enrichment of inflammatory, stress-response, and effector-associated pathways in ALS. (**C**) Volcano plot showing differentially expressed genes in γδ T cells from ALS versus healthy samples. Representative genes are labeled. (**D**) GSEA of γδ T-cell transcriptional programs. Positive NES values indicate pathways enriched in ALS. (**E**) Volcano plot showing differentially expressed genes in MAIT cells from ALS versus healthy samples. Representative genes are labeled.

Because our analyses pointed to enrichment of innate-like lymphocytes, we next examined their transcriptional states in greater detail. Among all immune populations profiled, NK cells exhibited the most extensive transcriptional remodeling. ALS-associated NK-cell genes included *RPL17*, *EEF1G*, *TLE5*, *TSC22D3*, *SNHG6*, *SNHG5*, *SNHG29*, *GAS5*, *SEPTIN9*, *ATP6V0C*, *LINC*-*PINT*, *MIF*, *JUN*, *DUSP1*, *NFKBIA*, *PMAIP1*, and *S100A4* transcripts, together with multiple ribosomal and stress-response transcripts (Fig. 5A). This broad signature is consistent with a highly activated NK cell state characterized by increased inflammatory signaling and effector readiness.

Gene-set enrichment analysis further emphasized the breadth of NK-cell activation. ALS NK cells were enriched for hypoxia, IFN-γ response, TNF-α/NF-κB signaling, EMT, interferon-α response, reactive oxygen species, KRAS signaling, apical junction, apoptosis, p53 pathway, and G2M checkpoint (Fig. 5B). γδ T cells also displayed a clear ALS-associated program, with increased *TLE5*, *GNLY*, *EEF1G*, *MT2A*, *SNHG5*, and *RPL17* transcripts, and pathway enrichment for hypoxia, TNF-α/NF-κB signaling, EMT, KRAS signaling, mTORC1 signaling, IFN-γ response, xenobiotic metabolism, apoptosis, and p53 pathway (Fig. 5C, D). MAIT cells showed a related but somewhat narrower response, including increased *NFKBIA*, *RPL36A*, *RPL17*, *SEPTIN7*, *TLE5*, *GAS5*, *SNHG6*, and *GNLY* transcripts (Fig. 5E). Together, these findings indicate that ALS preferentially remodels innate-like lymphocyte compartments, establishing a broad inflammatory axis centered on NK cells and γδ T cells and extending to MAIT populations.

### NK and γδ T cells contribute to the systemic inflammation in ALS patients.

**Figure 6:**
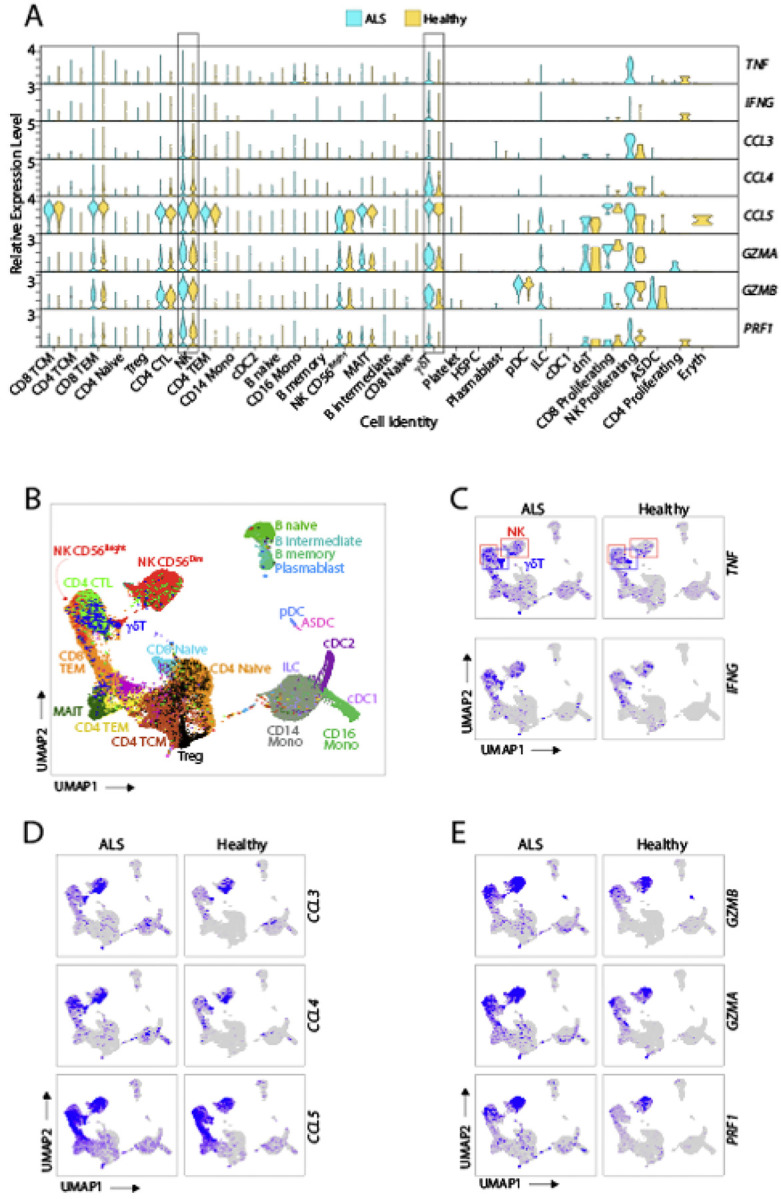
NK cells and γδ T cells are dominant sources of inflammatory chemokine and cytotoxic effector transcripts in ALS. **(A)** Distribution of selected inflammatory cytokine, chemokine, and cytotoxicity-associated transcripts across annotated immune populations in ALS and healthy samples, including TNF, IFNG, CCL3, CCL4, CCL5, GZMA, GZMB, and PRF1. (**B**) UMAP of the high-resolution PBMC annotation used for feature visualization in panels (C–E). (**C**) Feature plots showing TNF and IFNG transcript expression in ALS and healthy samples. (**D**) Feature plots showing chemokine transcript expression (CCL3, CCL4, and CCL5) in ALS and healthy samples. (**E**) Feature plots showing cytotoxic effector transcript expression (GZMB, GZMA, and PRF1) in ALS and healthy samples.

Finding the significant upregulation of IFN-γ and TNF-α signaling pathways among the immune populations, we further examined the inflammatory genes involved in these pathways and compared them between patient groups (Fig. 6A). Among the specified cell types, NK cells exhibited the highest expression of chemokines and cytotoxicity genes, with a notable increase in ALS patients. γδ T cells in ALS patients showed a significant increase in cytokines, chemokines, and cytotoxicity genes compared to the healthy group (Fig. 6A). Further gene expression analysis using UMAP revealed that T cells and NK cells are major contributors to IFNG and TNF expression in ALS patients (Fig. 6B and 6C). Chemokine signals, including *CCL3*, *CCL4*, and *CCL5* transcripts, are exclusively enriched in γδ T cells in ALS patients (Fig. 6D). Cytotoxicity genes, including *GZMB*, *GZMA*, and *PRF1*, are universally increased in the NK and CD8^+^ T cell compartments in ALS patients (Fig. 6E). With pro-inflammatory genes upregulated across NK and γδ T cells, these cells may be contributing to ALS disease progression.

### NK and γδ T cells emerge as dominant sources of inflammatory chemokine and cytotoxic signatures in ALS

Given the repeated enrichment of TNF-α/NF-κB, IFN-γ, and stress-response pathways across multiple immune populations, we next asked which cell types most strongly contributed to these inflammatory outputs. Comparative analysis of cytokine-, chemokine-, and cytotoxicity-associated transcripts identified NK cells and γδ T cells as the most prominent effector populations in ALS (Fig. 6A–E). *TNF* and *IFNG* transcript expression localized predominantly to T/NK compartments and appeared stronger and broader in ALS than in controls (Fig. 6B, C). Likewise, *CCL3*, *CCL4*, and *CCL5* transcripts were concentrated in innate-like effector populations, with particularly strong enrichment in γδ T cells from ALS samples (Fig. 6D).

In parallel, canonical cytotoxicity-associated genes, including *GZMB*, *GZMA*, and *PRF1*, were most strongly represented in NK cells and cytotoxic T cells and were increased in ALS patient samples (Fig. 6E). These data indicate that the ALS PBMC landscape is not uniformly inflamed in a nonspecific manner; rather, it is disproportionately shaped by innate-like lymphocyte populations with strong cytokine, chemokine, and cytotoxic potential. NK cells appear to provide the broadest combined inflammatory and cytotoxic module, whereas γδ T cells contribute especially strong chemokine-associated inflammatory output.

## Discussion

Our integrated genomic and single-cell analyses support a model in which ALS is associated with a structured peripheral immune-inflammatory state. We proposed three mechanistic inferences from our data. First, the genomic findings support a multi-hit susceptibility architecture in which shared background variants coexist with patient-enriched alterations. Second, single-cell data indicate that ALS is associated with a systemic stress-imprinted immune transcriptome distributed across multiple PBMC compartments, consistent with RNA/proteostasis-linked dysfunction. Third, the inflammatory and cytotoxic output of this remodeled immune landscape is concentrated in NK cells and γδ T cells, which emerge as the dominant innate-like effector populations in ALS. This framework provides a mechanistic structure linking inherited susceptibility to peripheral immune remodeling (8, 46).

### A genetic multi-hit susceptibility may establish a permissive immune state for ALS pathology.

The twin-based genomic analysis argues against a single-gene explanation for disease manifestation. The affected twin carried private or enriched variants in genes with established or plausible relevance to ALS biology, including *TARDBP*, *ERBB4*, *PRF1*, *ANG*, *SPG11*, *ATXN2L*, and *FUS*. In contrast, the unaffected twin carried private variants in canonical ALS-associated genes such as *C9orf72* and *HNRNPA1*. In addition, multiple genes contained shared background variants with twin-specific variants, including *ALS2*, *PRPH2*, Fig. 4, *SETX*, *TRPM7*, *UNC13A*, and *NEFH*. This pattern is most consistent with a multi-hit or modifier-rich model, in which penetrance depends on the cumulative burden and combination of variants rather than on any single mutation alone. That interpretation fits well with the broader ALS genetics, which increasingly recognizes oligogenicity, incomplete penetrance, and context-dependent disease expression as central features of the disorder (6–8). Importantly, the patient-enriched variants map to at least two functionally distinct axes: a) genes linked to RNA metabolism, stress signaling, and neurodegenerative vulnerability, including *TARDBP*, *FUS*, *ANG*, and *ATXN2L*; and b) a gene linked to cytotoxic immune function, such as *PRF1*. Currently, we do not know whether the missense mutation in the *PRF1* gene increases or decreases its protein stability and function. However, this suggests that inherited susceptibility in ALS may influence both neuron-intrinsic vulnerability and alterations in immune-effector function.

### ALS is associated with a systemic stress-imprinted immune state.

A second, non-mutually exclusive mechanism suggested by our data is that ALS-associated genetic variants are linked to a systemic stress-adapted immune transcriptome rather than a defect restricted to motor neurons (12). Across multiple PBMC compartments, including CD4^+^ T cells, CD8^+^ T cells, B cells, dendritic cells, monocytes, and NK cells, we observed recurrent enrichment of hypoxia-associated pathways, reactive oxygen species, p53-linked stress programs (47), ribosomal remodeling, and inflammatory modules centered on TNF-α/NF-κB and IFN-γ. This pattern was accompanied by repeated induction of *TSC22D3* and *SNHG*-family noncoding RNAs across several immune subsets (11). Together, these findings suggest that the peripheral immune system in ALS is not simply activated, but transcriptionally conditioned by chronic stress.

This interpretation is particularly compelling in light of the patient-enriched variants in *TARDBP*, *FUS*, *ANG*, and *ATXN2L*. Although the present study does not establish direct causality between these variants and immune-cell transcriptional states, the data are consistent with the idea that RNA metabolism and proteostatic dysfunction may have systemic consequences extending beyond neurons (45). The recurrent appearance of *SNHG*-family transcripts and broader stress-response pathways across immune subsets strengthens this argument by suggesting that post-transcriptional dysregulation may be embedded within the peripheral immune phenotype of ALS. An important implication of this model is that the ALS immune phenotype is broad but not uniform. Different immune lineages appear to interpret a common systemic stress signal in line with their lineage-specific biology. CD4^+^ and CD8^+^ T cells acquire inflammatory and stress-adapted programs; B cells display activation coupled to compensatory regulation; dendritic cells align more closely with inflammatory signaling; and monocytes occupy a more mixed stress-metabolic state. Thus, the shared stress-imprinted program is distributed across the immune system, but its downstream expression remains cell-type dependent.

### NK cells and γδ T cells define the dominant inflammatory genotype in ALS.

The most striking cellular conclusion of this study is that the inflammatory output of the ALS immune landscape is not diffuse across PBMCs, but instead predominantly present in innate-like cytotoxic effectors, particularly NK cells and γδ T cells (31). NK cells exhibited the broadest transcriptional remodeling of any immune population, with strong enrichment of inflammatory, stress-response, and effector-associated pathways, including TNF-α/NF-κB, IFN-γ response, hypoxia, reactive oxygen species, apoptosis, interferon-α response, and p53-linked stress programs. γδ T cells also showed a clear ALS-associated inflammatory program, with especially strong enrichment of chemokine-associated output. When effector transcripts were directly compared across cell populations, NK cells and γδ T cells emerged as the dominant sources of TNF-α, IFN-g, and CCL3/4/5, whereas NK cells and CD8^+^ T cells carried the strongest GZMB, GZMA, and PRF1 signatures (28).

These observations support a third mechanistic inference. NK cells and γδ T cells function as the principal inflammatory amplifiers within the remodeled ALS immune landscape. NK cells appear to provide the comprehensive combined cytokine, chemokine, and cytotoxic module. In contrast, γδ T cells appear particularly enriched for the CCL3/CCL4/CCL5 axis and may therefore be especially effective at amplifying leukocyte recruitment and inflammatory crosstalk. NK cells are increasingly recognized as heterogeneous (36) and transcriptionally plastic (46). In contrast, γδ T cells occupy a unique position at the interface of innate and adaptive immunity and can respond rapidly. In the context of ALS, the combined prominence of these two populations suggests that peripheral inflammation is organized around a dominant cytotoxic innate effector circuit rather than a generalized activation of all immune lineages. We therefore favor a model in which ALS-associated genetic susceptibility not only predisposes to neurodegeneration but also licenses a peripheral innate inflammatory transcriptomic state, executed predominantly by NK cells and γδ T cells.

### An integrated model for peripheral immune remodeling in ALS.

Taken together, our data support an integrated model with three linked layers. First, multilayered genetic susceptibility establishes a permissive background rather than a single deterministic lesion. Second, variants linked to RNA metabolism, neurodegeneration, and stress biology are associated with a systemic stress-imprinted immune transcriptome distributed across PBMC subsets. Third, the inflammatory and cytotoxic output of this remodeled landscape is funneled predominantly through NK cells and γδ T cells. In this framework, ALS-associated immune remodeling is not random. It is shaped by inherited susceptibility, translated into a broad stress-adapted immune state, and executed through a dominant innate effector program. This integrated model also helps reconcile several observations that might otherwise appear disconnected. The repeated appearance of ribosomal genes, *TSC22D3*, and *SNHG*-family transcripts across multiple lineages can be understood as the distributed imprint of chronic systemic stress and altered RNA regulation. The enrichment of innate-like lymphocyte subsets can be understood as a selective remodeling of compartments poised for rapid effector output. And the prominence of transcripts encoding TNF-α, IFN-g, CCL3/4/5, GZMB, GZMA, and PRF1 in NK and γδ T-cell compartments can be understood as the downstream execution arm of this broader immune reprogramming in ALS patients.

### Translational implications and limitations.

The translational implications of these findings are twofold. First, the data identify NK cells and γδ T cells as candidate peripheral cellular biomarkers of ALS-associated immune activation. Because these populations display the most integrated cytokine-, chemokine-, and cytotoxicity-associated signatures, they may provide more informative blood-based readouts than bulk PBMC analyses (34). Second, they identify innate lymphocytes as candidate immunoregulatory targets. This does not imply that NK cells or γδ T cells are proven drivers of motor neuron degeneration in patients (27); the present study remains transcriptional and associative. However, the convergence of compositional enrichment, pathway activation, and effector-molecule expression in these populations makes them compelling candidates for mechanistic and translational follow-up. We acknowledge several limitations. The cohort size is modest, especially for single-cell analysis, and the discordant twin genomic comparison is hypothesis-generating rather than definitive. The sequencing was performed on PBMCs rather than affected tissues, so these data capture the peripheral immune landscape rather than the full neuroimmune microenvironment. In addition, transcript abundance does not establish protein production, secretion, or function. Thus, although transcripts encoding TNF-α, IFN-g, CCL3/4/5, GZMB, GZMA, and PRF1 strongly implicate NK and γδ T cells in the ALS inflammatory milieu, these data alone do not prove direct pathogenicity. Functional validation in larger cohorts, ideally including protein profiling, cytokine-release assays, cytotoxicity assays, and neuron-immune co-culture systems, will be required to establish causality and therapeutic tractability.

In summary, our study supports a model in which ALS is associated with genetically heterogeneous but mechanistically convergent peripheral immune remodeling. The genomic data support a multi-hit susceptibility architecture. The single-cell data identify a stress-imprinted immune state distributed across PBMC subsets. And within that landscape, NK cells and γδ T cells emerge as the principal inflammatory-cytotoxic effectors. Together, these findings define ALS as a multi-hit immuneneurodegenerative disorder in which RNA/proteostasis-linked genetic susceptibility establishes a stress-imprinted immune transcriptome and drives a dominant NK/γδ T-cell inflammatory-cytotoxic circuit.

## Materials and Methods

### ALS sample collection

Written informed consent was obtained from healthy donors and patients diagnosed with amyotrophic lateral sclerosis (ALS) under a protocol approved by the UCLA Institutional Review Board (IRB # 11–000781). All procedures were conducted in accordance with UCLA-IRB approval. ALS diagnoses were established by the treating neurologists according to standard clinical criteria by the South Florida Bone Marrow Stem Cell Transplant Institute, DBA Maharaj Institute of Immune Regenerative Medicine. For paired genetic analysis, peripheral blood was collected from one patient with ALS and his monozygotic twin brother by the South Florida Bone Marrow Stem Cell Transplant Institute, DBA Maharaj Institute of Immune Regenerative Medicine. N-acetylcysteine (NAC) treatment, when present, was administered as part of routine clinical care at the discretion of the treating physician at the South Florida Bone Marrow Stem Cell Transplant Institute, DBA Maharaj Institute of Immune Regenerative Medicine. Patient received weekly NAC infusions.

### PBMC isolation and cryopreservation

Peripheral blood mononuclear cells (PBMCs) were isolated from whole blood by density-gradient centrifugation as described previously (48). Isolated PBMCs were either used immediately for downstream applications or cryopreserved in 90% fetal bovine serum (FBS) and 10% dimethyl sulfoxide (DMSO) in liquid nitrogen until analysis.

### Whole-genome sequencing

For whole-genome sequencing (WGS), PBMCs from the patient with ALS and his identical twin were lysed, and genomic DNA was extracted using a standard column-based protocol according to the manufacturer’s instructions. DNA yield and purity were assessed by spectrophotometry and fluorometric quantification, and high-molecular-weight DNA integrity was confirmed by agarose gel electrophoresis or equivalent fragment analysis before library preparation. Genomic DNA libraries were prepared by the Genomic Sciences and Precision Medicine Center at the Medical College of Wisconsin or at the University of California, Los Angeles, using an Illumina-compatible whole-genome library preparation workflow. Briefly, purified genomic DNA was mechanically fragmented to an average insert size of approximately 350 bp, followed by end repair, A-tailing, and ligation of dual-indexed Illumina sequencing adapters. Adapter-ligated fragments were size-selected and PCR amplified to generate the final sequencing libraries. Library fragment size distribution and concentration were confirmed by capillary electrophoresis and qPCR-based quantification. Pooled libraries were sequenced on an Illumina NovaSeq 6000 platform using paired-end 150-bp reads (2 × 150 bp), targeting an average depth of approximately 35× per genome. Base calling and demultiplexing were performed using the vendor’s standard pipeline to generate FASTQ files.

### WGS alignment, variant calling, and annotation

Sequencing reads were aligned to the human reference genome using the Burrows–Wheeler Aligner (BWA) (49). Following alignment, reads were sorted, and PCR duplicate reads were marked to reduce bias during variant discovery. Base quality score recalibration and germline variant calling were performed using the Genome Analysis Toolkit (GATK) (50). Single-nucleotide variants (SNVs) and small insertions/deletions (indels) were identified genome-wide, and direct comparison between the affected twin and the unaffected co-twin was performed to identify candidate variants associated with ALS status. Identified variants were subsequently annotated and filtered using SnpEff (51). Annotation included predicted coding consequence, affected transcript(s), predicted impact severity, and available clinical or population-frequency information. Variants considered low quality, including those with low depth, poor base quality, or strand bias, as well as those located in low-complexity or repetitive regions, were deprioritized. Common variants present at high allele frequency in population databases were also deprioritized. The remaining high-confidence rare or private coding variants were prioritized for biological interpretation, including genes with known or plausible relevance to neurodegeneration, inflammatory signaling, immune regulation, or cytotoxic lymphocyte function. This twin-based design, comparing an affected patient with ALS to his genetically matched unaffected monozygotic co-twin, enabled stringent filtering of shared germline background and facilitated prioritization of private or rare variants that could plausibly contribute to ALS susceptibility, disease progression, immune dysregulation, or differential responses to inflammatory stress.

### Single-cell RNA sequencing library preparation and sequencing

PBMCs from patients with ALS and healthy controls were processed for single-cell RNA sequencing using the 10x Genomics Chromium platform (36). Cells were loaded at a target capture of approximately 20,000 viable cells per sample. Single-cell cDNA libraries were generated using the Chromium Single Cell 3′ Reagent Kit v3.1 (10x Genomics) according to the manufacturer’s instructions. Libraries were sequenced on an Illumina NovaSeq 6000 platform to a depth of approximately 1500–2000 reads per cell. Following sequencing, raw base call files were demultiplexed, aligned to the human reference genome [reference genome/version], and unique molecular identifier (UMI) count matrices were generated using Cell Ranger v6.1.2 (10x Genomics) with default parameters. Downstream analyses were performed in R using Seurat v5.2.1.

### Quality control of single-cell RNA-seq data

Initial quality-control filtering was performed to remove low-quality cells and low-abundance genes. Genes detected in fewer than 5 cells were excluded. Cells with fewer than 200 or more than 4000–5000 detected genes were removed to reduce the inclusion of low-complexity droplets and potential multiplets. Cells were further filtered based on mitochondrial transcript content, excluding those with less than 1% or greater than 10% mitochondrial gene expression. Additional filtering criteria, including exclusion of cells with high hemoglobin gene expression and other low-quality populations, were applied as needed. After quality control, 33,667 high-quality cells were retained for downstream analysis.

### Data integration, clustering, and dimensionality reduction

Filtered samples were merged into a single Seurat object for integrated analysis. Cell-cycle scores were calculated using canonical S-phase and G2/M-phase gene sets and were included in downstream regression when appropriate. Data were normalized and variance-stabilized using SCTransform. Variables including UMI counts, mitochondrial transcript percentage, and cell-cycle scores were regressed during scaling to minimize technical effects. Dataset integration was performed using Seurat’s anchor-based integration workflow with variable features. Unsupervised clustering was carried out using the shared nearest neighbor (SNN) modularity optimization algorithm implemented in Seurat. Principal component analysis (PCA) was used for dimensionality reduction, and the top 30 principal components were selected for downstream clustering and uniform manifold approximation and projection (UMAP) visualization. Cluster resolution was selected based on clustree analysis and biological interpretability.

### Cell-type annotation

Cell identities were assigned using canonical immune cell marker genes and independently validated using the SingleR package v2.1, which performs reference-based annotation using transcriptomic profiles of purified cell populations. Broad immune lineages, including CD4^+^ T cells, CD8^+^ T cells, NK cells, B cells, monocytes, and dendritic cells, were first identified, followed by higher-resolution annotation of immune subsets including memory and effector T-cell states, γδ T cells, MAIT cells, NK-cell subsets, B-cell subsets, and myeloid populations.

### Differential gene expression and pathway analysis

Differential gene expression analysis between ALS and healthy control samples, or between annotated cell populations, was performed using the Wilcoxon rank-sum test implemented in the FindMarkers function in Seurat, unless otherwise specified. Genes detected in at least 3% of cells and with a minimum log fold change of 2 were considered for testing. P values were adjusted for multiple comparisons using the Benjamini–Hochberg method. Differentially expressed genes were visualized using volcano plots and used for downstream pathway analyses. Gene set enrichment analysis (GSEA) was performed on ranked differential-expression outputs to identify pathways enriched in ALS or healthy control samples within each immune subset. Hallmark gene sets from MSigDB v7.5.1 were used unless otherwise specified. Pathways with significant normalized enrichment scores and adjusted P values below [threshold] were considered enriched.

### Statistical analysis

Statistical analyses were performed using R with DESeq2 (v1.46.0) and GraphPad Prism v10, as appropriate. Data are presented as [mean ± SEM/median with interquartile range/box plots] as indicated in the figure legends. Statistical tests used for individual comparisons are described in the corresponding figure legends or analysis sections. A P-value less than 0.05 was considered statistically significant.

## Supplementary Material

Supplementary Files

This is a list of supplementary files associated with this preprint. Click to download.

- Table1.docx

## Data Availability

WGS and the single-cell RNA sequencing data will be submitted to the GEO database upon acceptance of the manuscript.
